# Literary Notes

**Published:** 1902-07

**Authors:** 


### LITERARY NOTES.

Poisons and Antidotes is the title of a useful card, lately issued by the Maltine Company, containing a list of the principal poisons, printed in color. Following each poisonous drug or substance is a group of most available and certain antidotes. It will serve as a reminder in emergencies, and should find a place on the office wall. It will be furnished on application to the publishers.

---

The Gleason Sanitarium, at Elmira, having reached the half-century time point, has issued a handsome brochure, which it has named a Fiftieth Year Anniversary Greeting. It contains some well-executed half-tone engravings and interesting text contributed from various sources. Dr. John C. Fisher continues his service as resident physician, which guarantees a safe and scientific medical administration.

---

Messrs. William Wood & Company, medical publishers of New York, have printed a new catalogue of their medical works that is illustrated by portraits of the principal authors. It is printed on highly finished book paper and is one of the handsomest medical catalogues that has yet been issued. It bespeaks the enterprise of this thriving house.

---

*The Southern California Practitioner*, which has been seventeen years in existence, presents a most flourishing appearance and is one of our exchanges that possesses great value. The June issue contains a symposium on tuberculosis that will be appreciated by every student of the subject. Dr. Walter Lindley, the editor, is to be congratulated upon the excellent quality of his magazine.

---

*American Gynecology* is the name of a new journal, which is announced to begin publication in July. It will be devoted to gynecology, abdominal surgery and obstetrics. It will be owned and controlled by a stock company consisting solely of members of the medical profession interested in its special field. Its editorial management is vested in a board made up as follows: J. Wesley Bovee, of Washington, D. C.; Charles Jewett, of New York; Charles P. Noble; of Philadelphia; Reuben Peterson, of Ann Arbor, Mich.; and J. Whitridge Williams, of Baltimore. Mr. E. W. Reynolds will be the business manager. The office of publication will be No. 1 Madison Avenue, New York.

---

Polk's Directory for 1902 has been issued. This is the seventh edition of this valuable work and contains 3,008 pages. It is one of the most valuable directories yet published and should find its way into the offices not only of physicians, but into every business house that has dealings with the medical profession. This edition is constructed on similar lines to its predecessors, but is very much enlarged and amplified, thus increasing its value though its price remains the same as heretofore—$10.00. Address R. L. Polk & Co., Detroit, Mich.

---

Fractures of the surgical neck of the humerus is the subject of Chart No. 8, issued by Battle & Co., of St. Louis. It gives in well drawn lines and striking colors the details of this quite common injury, and becomes a helpful reminder to the surgeon. These charts deserve preservation in album form.

